# Glycosylated gold nanoparticles in point of care diagnostics: from aggregation to lateral flow

**DOI:** 10.1039/d2cs00267a

**Published:** 2022-07-27

**Authors:** Alexander N. Baker, George W. Hawker-Bond, Panagiotis G. Georgiou, Simone Dedola, Robert A. Field, Matthew I. Gibson

**Affiliations:** Department of Chemistry, University of Warwick Gibbet Hill Road CV4 7AL Coventry UK m.i.gibson@warwick.ac.uk; Oxford University Clinical Academic Graduate School, John Radcliffe Hospital Oxford Oxford OX3 9DU UK; Iceni Glycoscience Ltd Norwich NR4 7GJ UK; Department of Chemistry and Manchester Institute of Biotechnology, University of Manchester Manchester M1 7DN UK; Division of Biomedical Sciences, Warwick Medical School, University of Warwick Gibbet Hill Road CV4 7AL Coventry UK

## Abstract

Current point-of-care lateral flow immunoassays, such as the home pregnancy test, rely on proteins as detection units (*e.g.* antibodies) to sense for analytes. Glycans play a fundamental role in biological signalling and recognition events such as pathogen adhesion and hence they are promising future alternatives to antibody-based biosensing and diagnostics. Here we introduce the potential of glycans coupled to gold nanoparticles as recognition agents for lateral flow diagnostics. We first introduce the concept of lateral flow, including a case study of lateral flow use in the field compared to other diagnostic tools. We then introduce glycosylated materials, the affinity gains achieved by the cluster glycoside effect and the current use of these in aggregation based assays. Finally, the potential role of glycans in lateral flow are explained, and examples of their successful use given.

## Abstract 2

Antibody-based lateral flow (immune) assays are well established, but here the emerging concept and potential of using glycans as the detection agents is reviewed.

## Graphical abstract

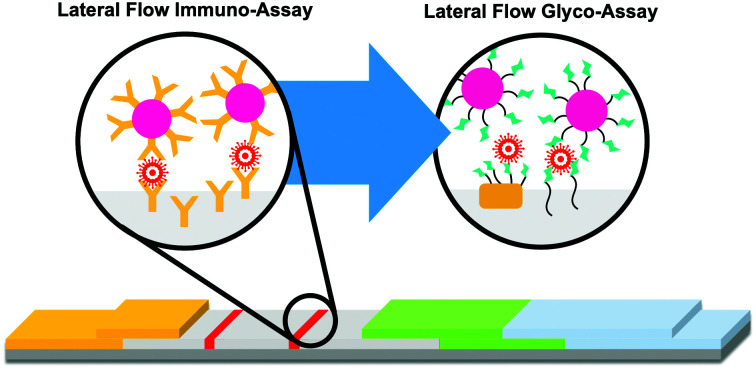

## 1. Introduction – point of care

The diagnosis of infectious diseases should be achieved by; determining infection sites, considering patient needs and gaining a microbiological diagnosis. In many cases, however, diagnosis is guided by symptomatic presentation rather than therapeutic approaches as many of these methods can take hours or days to produce a result. Observational approaches alone can in some cases reduce patient outcomes and the injudicious prescribing of broad-spectrum drugs which can contribute to antimicrobial resistance (AMR).^1^

The first point-of-care (POC) diagnostic was developed in 1962 to measure blood glucose levels.^2^ This was followed by the rapid pregnancy test *circa* 1969.^3,4^ Both of these diagnostics revolutionised care: allowing for testing and diagnosis by or with the patient present. In 2011, point-of-care tests (POCT), or “bedside testing” devices were worth $15 billion USD with a projected increase of 4% per annum.^5^

While there is no accepted definition for point-of-care testing, it can be considered to be rapid testing at, or near to, the point of need that is used to make a medical decision.^6–9^ Current POCT devices include the aforementioned glucose biosensor, dipsticks and lateral flow devices (LFDs);^10^ such as Malaria Rapid Diagnostic Tests (MRDTs)^11^ and pregnancy tests, that separate samples through a solid phase.^5^

**Fig. 1:**
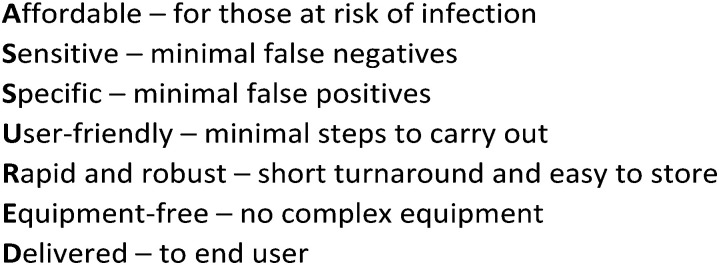
ASSURED acronym, WHO guidelines for the design of POCT.

Surveys of medical professionals, in relation to POCT, have indicated that sensitivity (> 90%) is considered the most important attribute, followed by a price of less than $20 USD and a short detection time.^12^ This is summarised by the World Health Organisation (WHO) guidelines for the design of POCT in the ASSURED acronym (Fig. 1).^13^

The cost-effectiveness of POCT, compared to traditional laboratory techniques, has been demonstrated in the UK National Health Service (NHS) Health Checks, an early detection tool for cardiovascular disease;^14^ a disease which costs the NHS approximately £7 billion GBP per year.^15^ The total expected costs (TEC) per completed Health Check was markedly reduced for POCT compared to laboratory techniques – £18 GBP per completed Health Check compared to £25 GBP, respectively, and attendance rates were higher for POCT.^14^

It is equally important to consider the perspectives of patients and health care professionals (HCPs) to POCT. During an increase of Dengue fever cases in Singapore, POCT were found to be more convenient, and marginally better than non-POCT in improving the speed and accuracy of diagnoses.^16^

The alternative to POC testing is (highly accurate) laboratory-based testing, which require trained staff, embedded health infrastructures and specialised facilities. This makes lab-based methods, considered standard in wealthier nations, cost-prohibitive in low- and middle-income countries. This is evidenced by the COVID-19 (caused by SARS-COV-2) pandemic. PCR-based approaches have been the primary tool for diagnosis but is not ideal for mass testing due to price, turnaround time and the need for centralised infrastructure.^17,18^ It is estimated that laboratory-independent POCT for four common infections (syphilis, malaria, tuberculosis and bacterial pneumonia) could prevent more than 1.2 million deaths per annum in low- and middle-income countries.^4^ Chromatographic lateral flow paper-based immunoassays, as highlighted by MRDTs and pregnancy tests, are of particular interest as POCT because of their low cost, speed of use, laboratory-independence and wide range of devices and analytes currently in use.^19^

This review article introduces the potential of glycans (sugars/carbohydrates) to be deployed in rapid, lateral flow assays, compared to the currently used antibody-based LFDs. We first summarise the chemical basis of POC lateral flow immunoassay (LFIA) diagnostics that are based upon antibodies. We then provide a discussion on how a lateral flow assay has been deployed in the real world. Finally, we discuss glycosylated materials, in particular glycosylated gold nanoparticles. We focus on gold particles as these are the basis of commercial tests, however other material cores are highlighted. We cover the rapidly emerging literature showing the development of paper-based lateral flow glyco-assays, LFGA.

## 2. Lateral flow – an overview

Lateral flow devices (LFDs) typically employ antibodies as sensing components; these devices are often termed lateral flow (immuno) assays (LFIA), or “sol particle immunoassays” as originally termed by Leuvering *et al.*^20^ LFIAs have been developed for a variety of targets, including; complement;^21^ anabolic steroids;^22^ prostate-specific antigen;^23^ leprosy^24^ and meningitis^25^ in serum; evidence of kidney injury in urine;^26^*V. cholerae* in faeces;^27^ and other marker proteins^28,29^ or pathogens^30^ in whole blood samples.^31^ This ability to target a broad range of analytes has led to the use of lateral flow devices in policing illicit drug use, for example.^32,33^

**Fig. 2:**
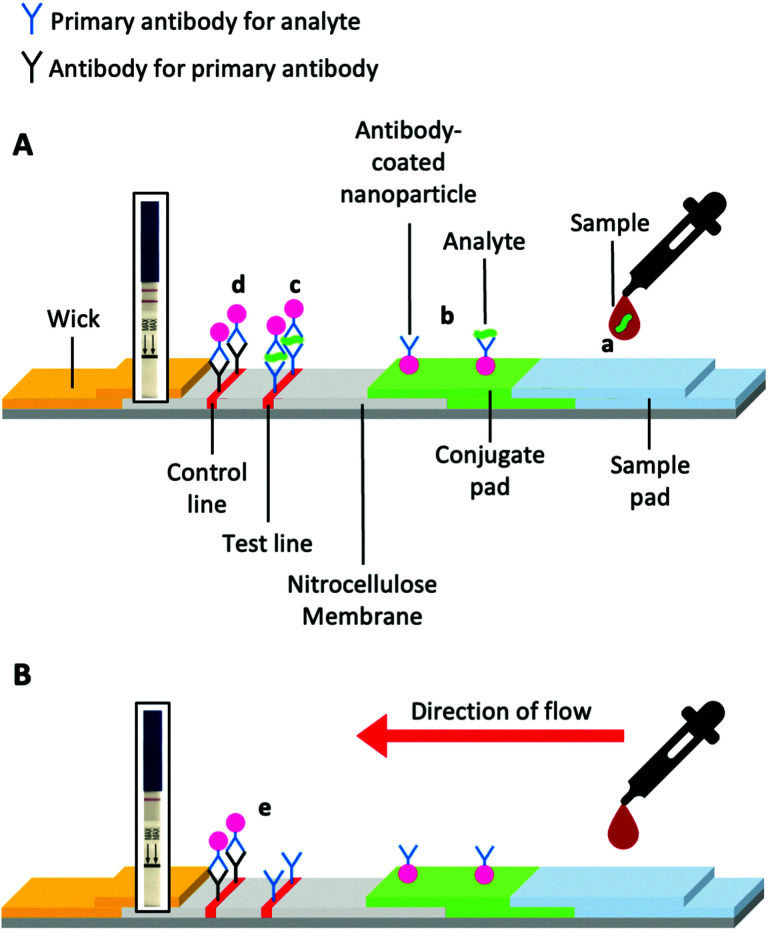
Schematic of a prototypical lateral flow device. (A) Device constituents in a successful positive test and (B) a successful negative test. Generic pregnancy tests for HCG detection inset.

LFDs typically contain two components; a mobile phase and a stationary phase. When a sample is applied to the strip's sample pad, it is drawn by capillary forces caused by the wick, through the device (Fig. 2A). Then the sample passes through the conjugate pad. If the sample contains the target analyte (Fig. 2Aa), it will bind antibody-coated gold nanoparticles (or other coloured nanomaterials) within the conjugate pad (Fig. 2A). The sample and nanoparticles (both bound and unbound) pass into the nitrocellulose membrane, where they move through two lines of the deposited material. One of these lines is a control line that containing antibodies specific to the primary antibody in the conjugate pad (Fig. 2Ad). Binding of the nanoparticles only to the control line indicates a successful test, but a negative sample (Fig. 2B). The test line contains antibodies specific to the analyte. A line will form if the analyte is “sandwiched” between antibodies immobilised in the test line and antibodies immobilised on the nanoparticles (Fig. 2Ac).

There are examples of multiplexed lateral flow tests.^34^ This multiplexed approach allows for greater efficiency and greater diagnostic precision and may reduce overall cost when tests are combined into one device. An earlier form of the lateral flow test, called a flow-through assay, utilised an immobilised sample as a test line – these devices are often less sensitive than lateral flow devices. However, they are employed when lateral flow is not possible for economic or chemical reasons or during the prototyping and the development stages of LFD design.^5,35^

## 3. The components of lateral flow

### The stationary phase

The stationary (immobile) phase comprises a receptor bound to the surface, usually an antibody. Larger antibodies do not require an immobilisation agent, with immunoglobulin G having an absorption of > 100 μg cm^−2^ on nitrocellulose; however, protein absorption can decrease with decreasing molecular weight.^36^ Bovine serum albumin (BSA), or less commonly ovalbumin, are used as carriers/immobilisation agents for small molecules such as nucleotides and small proteins.^19,37^ Less common immobilisation strategies include covalent epoxide–thiol and using fusion-proteins.^38^ Strategies to immobilise polynucleotides have also been reported.^39,40^

The low absorption of unconjugated antibodies was noted in polymeric systems by Aoyama *et al.*^41^ To overcome this they produced high surface area microcone architectures on polycarbonate sheets. The increased surface area enabled high antibody immobilisation levels. This step was inspired by the use of plastic and paper microfluidic systems,^42^ a rapidly growing technique that has been used to sense Ebola virus RNA,^43^*Salmonella typhimurium*^44^ and glucose.^45^

Studies with cellulose paper have also exploited biotin and the ability to covalently modify the cellulose surface for easy test line immobilisation.^46,47^ Tanaka *et al.* and Nagatani *et al.* have utilised the localised surface plasmon resonance (LSPR) phenomenon of gold nanoparticles in the stationary phase to enhance the signal-to-noise ratio when sensing for human chorionic gonadotrophin (HCG) and prostate-specific antigen respectively.^23,48^ This approach allowed for detection limits of 1 pg ml^−1^ of HCG,^48^ the hormone detected in the standard pregnancy test.

Reverse immunoassays that utilise “proteinticles” (protein-coated nanoparticles) in the stationary phase have also been developed.^49^ In these systems, antibodies against HIV and hepatitis A and B were tested in the mobile phase. However, the patient's immune system must mount an adaptive immune response for the test to be successful. Other tests, such as the Epstein-Barr Virus Antibody Test, also sense for an immune response to the pathogen. Although not POC, it does provide precedence for reverse immunoassay approaches.^50^

### The mobile phase

Nanoparticles for biosensing have received significant attention due to their unique optical properties, making them ideal for sensing activities,^51^ and their tunability in terms of morphology and composition.^52^ Plasmonic, especially gold, particles are the most common signal generating units. Plasmonic nanoparticles are intensely coloured because their free electron density can couple with electromagnetic photons with a wavelength greater than the size of the nanoparticle.^53–55^ The colour of a nanoparticle is not only dependent on its size but also its shape and the dielectric constant of the metal and medium it is in, as shown by the Fröhlich condition.^56–58^ Only light resonant to electron oscillation can excite the localised surface plasmon and be absorbed by it; this absorption leads to localised surface plasmon resonance (LSPR), producing the distinctive colour of the nanoparticle system, which in the case of gold is from red to blue. Plasmonic nanoparticles have high molar attenuation (extinction) coefficients (ε), hence their use in LFDs. For example, 35 nm gold nanoparticles (AuNPs) and silver nanoparticles (AgNPs) have attenuation coefficients far higher than common organic dyes such as Sudan III, methylene blue and crystal violet. The best porphyrin dyes, such as 5,10,15,20-tetra-21*H*-23*H*-porphine zinc, also have lower attenuation coefficients than AuNPs (Table 1). High attenuation coefficients lead to bright, vivid colours even at low concentrations in nanoparticles systems, making them ideal for sensing applications.

**Table 1:** Attenuation coefficients of a selection of nanoparticles and dyes

Chromophore	Molar attenuation coefficient (*ε*, M^−1^ cm^−1^)	Wavelength, *λ*_MAX_ (nm)
Gold nanoparticles, 35 nm^59^	∼6.1 × 10^9^	506
Silver nanoparticles, 30 nm^60^	∼1.5 × 10^10^	406
Sudan III^61^	∼3.0 × 10^4^	512
Methylene blue^61^	∼4.1 × 10^4^	654
Crystal violet^61^	∼7.6 × 10^4^	590
5,10,15,20-tetra-21*H*-23*H*-porphine zinc^61^	∼5.7 × 10^5^	422

Gold nanoparticles (AuNPs, 15–800 nm) are the most widely used plasmonic particles^31^ due to their biocompatibility, simple synthesis, low cost (at the concentration used) and straightforward functionalisation with organic molecules, particularly thiols. The most common method for AuNP synthesis uses HAuCl_4_, and sodium citrate as both a reducing and capping agent.^62–64^ By using seeded growth, anisotropic nanocrystals, such as rods, wires and triangles can be obtained and used in lateral flow systems.^65^

**Fig. 3:**
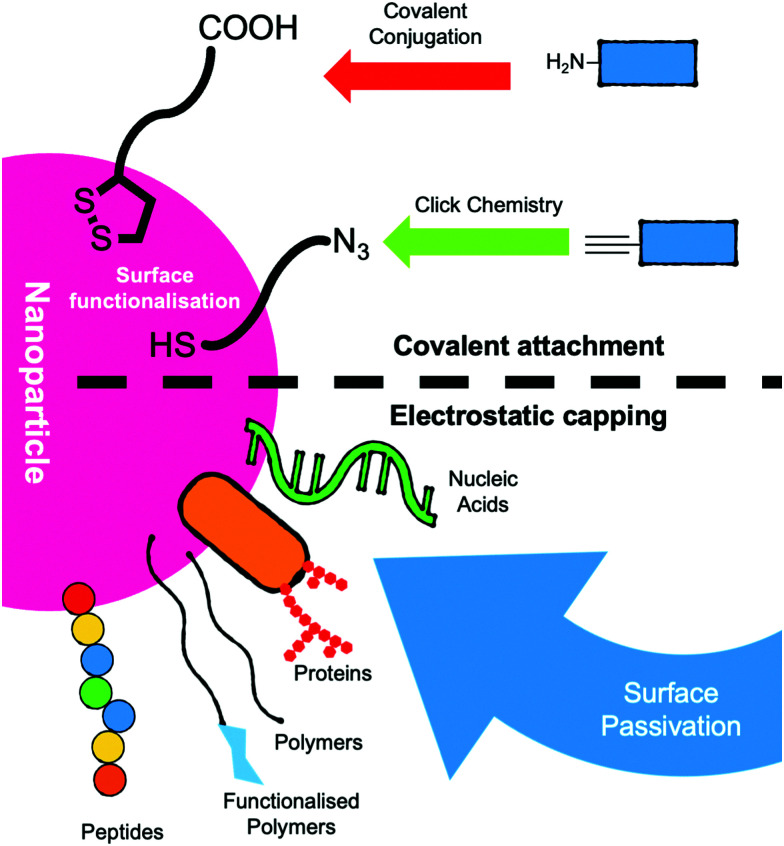
Schematic of strategies for the functionalisation of nanoparticles for use as the mobile phase in lateral flow devices.

AuNPs can be surface-modified with the analyte targeting components by surface passivation using sulphur-linked (for example, thiols and thioctic acids)^66^ or electrostatically charged antibodies or DNA strands for example.^39^ Capping ligands include; zwitterions, polymers, peptides, proteins, sugars and nucleic acids (Fig. 3).^51^ The vast majority of lateral flow systems utilise antibody capping groups and have been extensively reviewed.^19,31,37^

In addition to gold, but outside of the scope of this review, other nanoparticles have also been tested in lateral flow systems, including; core–shell gold nanoparticles,^67^ quantum dots,^68^ graphene oxide^69^ and carbon nanotubes.^70^ Furthermore, Dou *et al.* have produced nanomaterial-free antibody systems^71^ using crystal violet-stained antibodies, enabling a test to run in 14 minutes.

While many lateral flow devices are designed for qualitative analysis, often by eye, it is possible to quantify analyte concentrations. Urusov *et al.* describe three methods for quantitative detection; optical detection using a camera, scanner, or smartphone etc and image analysis software, or magnetic labels or electrically conductive labels.^72^ In these methods the intensity of the test line is used as an indication of the analyte concentration, *versus* a calibration curve. The benefits of quantification, as described by Urusov *et al.* include the removal of human subjectivity, a lower limit of detection and an understanding of the target's concentration. However, the use of an additional device for analysis does decrease the applicability of some setups in low-resource scenarios.

The simplicity, diversity and broad applicability to many targets have made lateral flow systems an attractive diagnostic. Many other POCT devices and lab-based diagnostics have been designed for use in more economically developed countries (MEDCs) but are not compatible in less economically developed countries (LEDCs).^73,74^

## 4. Malaria rapid diagnostic tests – a case study

### MRDTs in the field

**Fig. 4:**
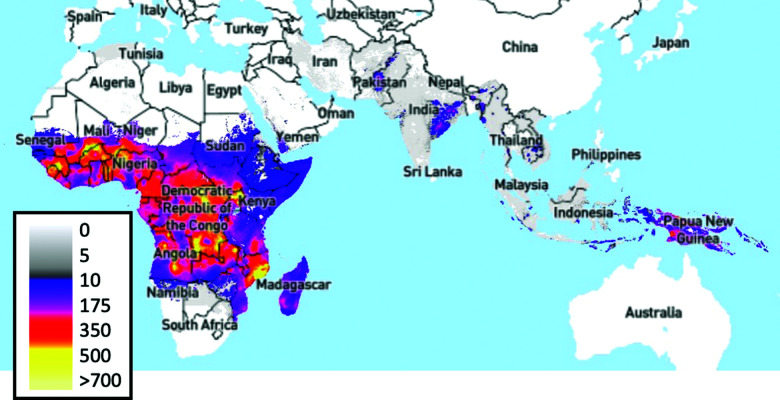
*Plasmodium falciparum* incidence per 1,000 people, 2017.^88,89^ Image produced using The Malaria Atlas Project, (Malaria Atlas Project, https://malariaatlas.org, (accessed 6 August 2019)).^90^

Malaria Rapid Diagnostic Tests (MRDTs) have been deployed in low- and middle-income countries as a successful example of the benefits and drawbacks of lateral flow compared to other POC devices. The World Health Organisation (WHO) estimated 438,000 deaths from malaria in 2014, with 90% of these deaths occurring in sub-Saharan Africa (Fig. 4).^30,75^ In 2001, artemisinin-based combination therapies (ACTs) were recommended by WHO as first-line treatments.^76^ Within the decade (2009), resistance to first-line ACT treatments was observed in the Greater Mekong Sub-region.^77–79^ Resistance to ACTs has not been observed in Africa. However, there is concern that resistance will be observed.^80,81^ During the period from 2008–2015, 35% of suspected malaria cases in Africa were not confirmed with diagnostic tests, which led to antimalarial overuse, increasing the risk of ACT resistance.^30,81,82^ In the same period, resistance was further compounded by broader economic issues. Most malaria-affected countries use treatments costing less than $0.5 USD per capita, while artemisinin-based combinatorial therapies cost ∼$5 USD per capita.^83–85^ A shift to a diagnostics-led approach could alleviate this problem, with research in Nigerian community pharmacies demonstrating the cost-effectiveness of MRDT-led treatment.^86^ The United Nations Children's Fund (UNICEF) increased its procurement of Malaria Rapid Diagnostic Tests (MRDTs) from 3.8 million tests to 15 million.^87^

**Fig. 5:**
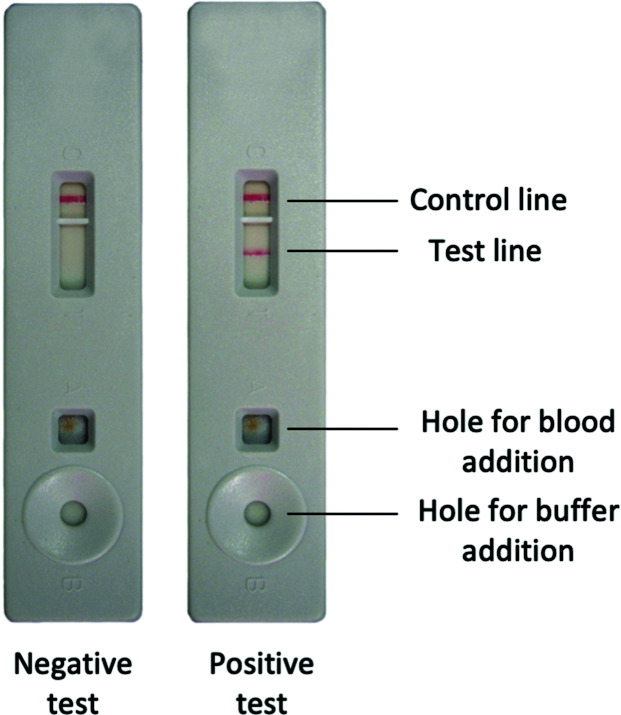
Image of negative and positive MRDT, taken from WHO documents.^91^

MRDTs are POC immunochromatographic or photometric lateral flow devices utilising peripheral blood samples and selenium dyes or AuNPs conjugated to monoclonal antibodies as sensing units, and an example test is shown in Fig. 5.

They are handheld POCT for malaria detection and can differentiate four out of five malaria species (*Plasmodium vivax*, *P. ovale*, *P. malariae* and *P. falciparum*). The devices are, however, insensitive to *P. knowlesi*: a simian parasite known to infect humans. MRDTs target a variety of antigen analytes such as histidine-rich protein 2 (HRP-2 is synthesised by all *P. falciparum* parasites as an abundant water-soluble protein, unlike HRP-1 and HRP-3),^92^*Plasmodium* lactate dehydrogenase (pLDH) and *Plasmodium* aldolase.^93,94^

### Alternatives to MRDTs and other lateral flow systems

MRDTs are less sensitive than alternative non-POCT techniques, such as microscopy of thick blood films and PCR techniques which can detect low parasite concentrations of 50 parasites per μL and 5 parasites per μL, respectively. While WHO does not test MRDTs at concentrations below 200 parasites per μL.^30,93^ MRDTs are also less capable of determining parasite species beyond *P. falciparum* and *P. vivax*, unlike Giesma-stained blood microscopy,^95^ the most common diagnostic technique, that can have a sensitivity of 95% and a specificity of 98%.^30,93,96^ Furthermore, this high sensitivity and specificity can be achieved at $0.2 USD per sample at a rate of one sample every 20 minutes.^96^ However, microscopy is a lab-based technique that requires an expert to carry out the analysis, plus the sensitivity can drop to 10% in the field.^97^

Due to the COVID-19 pandemic, diagnostics have been rapidly rolled out globally, with rRT-PCR (real-time reverse transcription-polymerase chain reaction) being the gold standard, but turn-around time can be a limiting factor. In July 2020, in the United States, the average wait time for a COVID-19 RT-PCR test was 4 days, and only 37% of people received results within 2 days.^98,99^ A comparison of rRT-PCR to LFDs showed that lateral flow device sensitivity was > 85% and its specificity > 95%, with a run time of just 15 minutes.^100^

Several other techniques have emerged to address the time and cost issues of (the very accurate) rRT-PCRs. Loop-mediated isothermal amplification^101^ (LAMP) can be run on a benchtop with various outputs generating the signal,^101,102^ in between 20–90 minutes.^101^ A LAMP device for malaria diagnosis was field-tested in India and Thailand and showed similar sensitivities and specificities to rRT-PCR. LAMP devices have also been developed for SARS-COV-2 and compared well to RT-PCR^103^ and for malaria diagnosis too.^104^ Aerts *et al.*,^105^ when discussing the cost-effectiveness of LAMP, microscopy and lateral flow tests in diagnosing cutaneous leishmaniasis in Afghanistan, suggested that all these diagnostic platforms have a role to play in different healthcare settings. They found that lateral flow systems reduce overall cost when used outside the clinic. Whereas microscopy is favourable in the clinic except in periods of high demand where LAMP and lateral flow devices are more cost-effective.

### MRDT summary

In summary, MRDT lateral flow systems are cost-effective, fast to run and do not require a specialist to perform nor a dedicated laboratory. MRDTs provide an interesting case study in applying POC lateral flow as a low-cost, fast diagnostic *versus* lab-based and more expensive POC systems in LEDCs. Moreover, studies carried out in MEDCs (the United States of America) have found that MRDTs can outperform blood smear microscopy,^106^ showing the potential of lateral flow systems in more economically developed countries where patients and health care professionals value short turnaround times.^5,12^ Therefore, while more expensive reusable POCTs and lab-based diagnostic systems have the potential to provide higher specificities, they do not have the same ease of use, minimal power needs, token maintenance requirements, and low costs of LFDs. It is essential to highlight, though, that some reports of MRDTs suggest they are still costly and challenging to use.^107^

Crucially, antibody-based MRDTs can degrade when not transported and stored at low temperatures (“cold-chained”), becoming less-sensitive in conditions common in the field^108^ – highlighting a potential weakness of protein-based LFDs and hence there is interest in developing antibody-free lateral flow devices.

## 5. Beyond antibodies: glycans as recognition units

Current POC diagnostics, particularly lateral flow systems (above), rely on antibodies as recognition units, owing to their high specificity and established methods for their production and nanoparticle conjugation. However, they are not the only biological recognition motifs; nucleic acids^109^ and lectins have also been incorporated but are still far less commonly used than antibodies.^110^ Glycans (carbohydrates) offer an alternative to these recognition units, and their study might bring new opportunities.

**Fig. 6:**
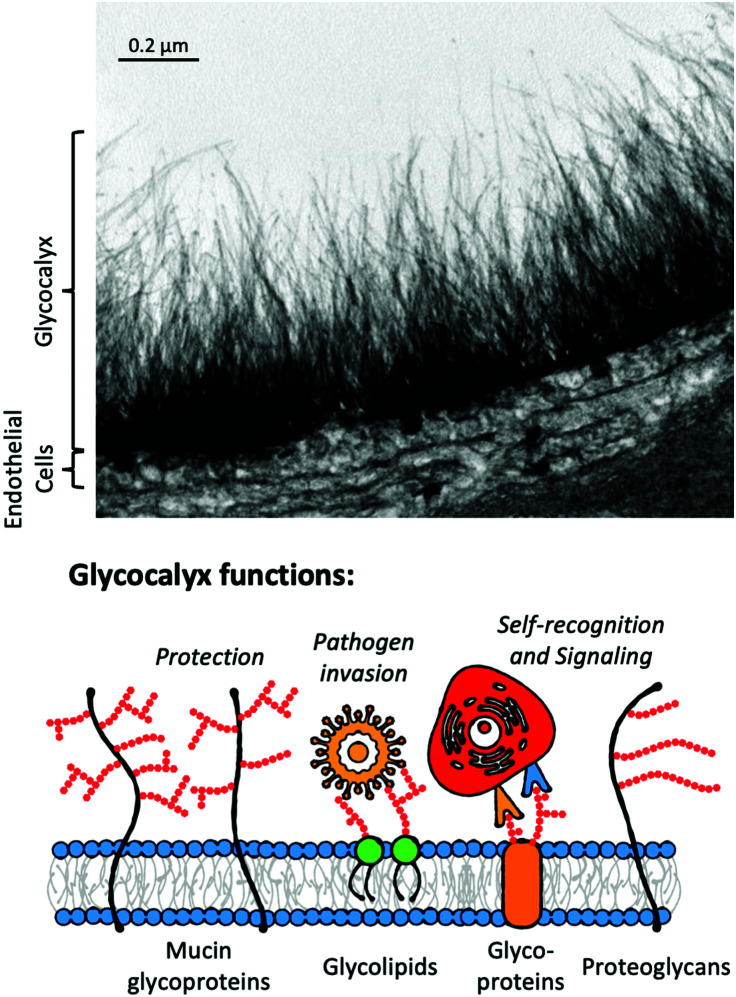
Electron micrograph of goat coronary capillary stained with Alcian blue and diagrammatic representation of the glycocalyx highlighting glycocalyx functions. Electron micrograph from van den Berg *et al.* reproduced from ref. 127 (Figure 75.1.) with permission from publisher Cambridge University Press, copyright 2007.

Glycans are ubiquitous in biological systems;^111^ with over half of all human proteins glycosylated.^112^ Glycosylation covalently adds glycans to proteins, lipids or other molecules (including RNA^113^) to form glycoconjugates in a dynamic process, regulated by glycosyl transferases and glycosidases.^114^ As it is not template driven, glycosylation cannot be (readily) predicted from genomic information alone. Glycoconjugates perform a vast range of roles, including; cell signalling,^115^ hormonal actions,^116^ cancer progression,^117^ correct protein folding/structure^118,119^ and mediating immune responses.^120^ Many of these processes occur at the glycocalyx (Fig. 6), found on the surface of eukaryotic and bacterial cells, providing the primary interface for cells with their external environment.^121^ The nature of glycan presentation on mammalian cells is a crucial marker of a disease state,^122,123^ influenza species specificity^124^ and influenza A viruses’ zoonotic potential.^125^ Antigens from *P. falciparum* have been shown to bind to sialyllactoses and other derivatives found on human erythrocytic glycoprotein A.^126^

Consequently, there is a significant opportunity to target the glycans, or the “glycan-readers” termed lectins, in diagnostics. Lectins are a broad family defined as glycan-binding proteins which are neither enzymes, transporters or antibodies and often have highly conserved carbohydrate-binding domains (CBD).^128,129^ Examples of lectins (or lectin-like proteins) relevant to healthcare include the Shiga toxins,^130^ cholera toxin^131^ and ricin,^132^ while also being found in snake venoms^133^ and biocides in marine algae.^134^ Notably, the use of lectins for staining histology samples has been established for decades.^128^

The use of lectins in biosensor design has been extensively reviewed.^135,136^ For example, Damborský *et al.* have reported a lectin-based LFD for prostate-specific antigen (PSA).^110^ There is extensive literature on the use of glycans in various applications, which is beyond the scope of this focussed review, but includes; anti-adhesion therapies,^137,138^ glyconanotechnology,^139,140^ antimicrobial applications^141^ and, influenza^142^ and human immunodeficiency virus (HIV)^143^ vaccine development. A challenge (and opportunity) lies in the synthesis of oligosaccharides, which inhabit a vast chemical space beyond that of other biological macromolecules of nucleic acid and protein origin.^144^ However, access to glycans for recognition studies is rapidly expanding,^145^ thanks to the emergence of automated glycan synthesis^146–148^ and the use of chemo-enzymatic strategies for greater glycan diversification.^149–151^

### Multivalency and glyco-materials

**Fig. 7:**
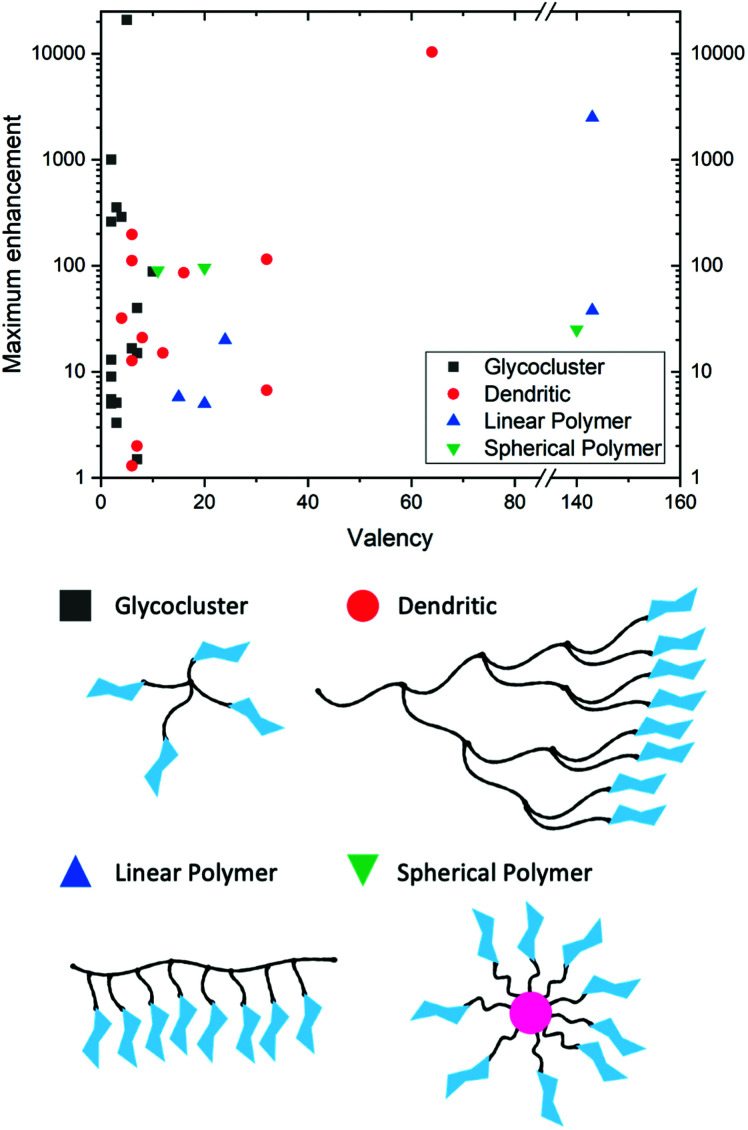
Graphical representation of a selection of glycoconjugates in “Table 1” from Lundquist *et al.*^152^ highlighting the maximum enhancement in binding (corrected for ligand valency) as valency changes.

Glycan–lectin interactions are typically weak with *K*_*d*_ (dissociation constants) ranging from mM to μM,^139,152^ which is significantly weaker than antibody–ligand interactions (in the nM to pM range). This is overcome in Nature by the presentation of multiple copies of glycans leading to statistical rebinding and chelation, resulting in significant enhancements to binding affinities – known as the “cluster glycoside effect”.^153,154^ The observed affinity enhancement (per glycan) as valency increases is non-linear and is dependent on the architecture of the glycan, its linker and its accessibility to the target. Due to this, there has been significant interest in using materials chemistry (polymers, particles and surfaces) to present multiple copies of glycans. In the context of diagnostic devices, the cluster glycoside effect is crucial to obtaining the necessary affinity and selectivity, which is not possible using individual glycans. To show the power of the cluster glycoside effect, Fig. 7 shows a summary of 37 glycoconjugates from a previous review.^152^ This simple representation highlights enhancement effects, ranging from 2 to >10 000-fold increases associated with multivalency. However, it also shows that the simplified assumption that ever larger, higher valency systems lead to increased affinity is not valid: the effects are more subtle. For example, one of the most potent inhibitors of the cholera toxin has just five precisely placed glycans.^155^ This is crucial to understand how glycosylated gold (or other) nanoparticles may apply in lateral flow diagnostics and how they can be designed more effectively.

## 6. Structural and synthetic strategies for glycan presentation

In order to integrate glycans into nanoparticles for LFDs, it is crucial to consider the synthesis (and glycan-binding potential) of multivalent glycosylated materials, which is essential to generate the needed affinity/avidity. Whilst current LFDs use gold cores, other nanomaterials can be employed, and it is crucial to also consider these routes to multivalency, including polymeric methods. It is important to note that beyond multivalency/presentation on polymeric scaffolds, glycan arrays have allowed the probing of multi-antennary/branched glycans.^156^ The benefits of this approach are well illustrated by Wang *et al.,* who synthesised a series of asymmetrically substituted multi-antennary glycans and found differential binding between the structures despite presenting similar terminal sugars.^157^

### Glycoclusters and dendrimers

Glycoclusters provide well defined and controlled glycan presenting architectures. André *et al.* designed a series of glycoclusters to increase affinity relative to the free sugar and enhance selectivity towards galectins, in some cases more than halving the IC_50_ in a tetravalent arrangement.^158^ More complex glycocluster systems have allowed linker length and valency tuning to target virulence factors such as LecA of *Pseudomonas aeruginosa*^159^ and the fimbriae-mediated adhesion of *E. coli*,^160^ respectively. Other groups have harnessed non-synthetic cores and directed evolution to design glycoclusters.^161^

Dendrimers are oligomeric or polymeric structures produced iteratively, forming tree-like, highly branched regular structures and have been used as platforms for glycodendrimers.^162,163^ A study by Woller *et al.* measured the binding enhancement realised as dendrimer generation increased in mannose-functionalised dendrimers and saw a 660-fold increase in activity per sugar, *versus* free sugar, when targeting Concanavalin A (ConA).^164^ However, glycodendrimers often present a compromise – higher valences are accessible at the cost of decreased structural control and more challenging syntheses, unlike in glycoclusters, where structural control is often favoured over high valences. The high tunability of dendrimers also makes them an attractive approach for sugar presentation, with void spaces in larger dendrimers providing room for molecular cargo, potentially for clinical applications.^165,166^

### Polymeric approaches

Glycopolymers present glycans either on their side chains or end-groups. Due to the relative ease of polymer synthesis, glycopolymers present a convenient route to engineering multivalency.^167,168^ Controlled radical polymerisation techniques have been crucial to glycopolymer development enabling the synthesis of macromolecules with predictable chain lengths, compositions, and architectures – essential to dissect the impact of multivalency. These techniques include atom transfer radical polymerisation (ATRP),^169^ reversible addition–fragmentation chain-transfer (RAFT)^170^ polymerisation, ring-opening metathesis polymerisation (ROMP)^171^ and nitroxide-mediated polymerisation (NMP).^172^

**Fig. 8:**
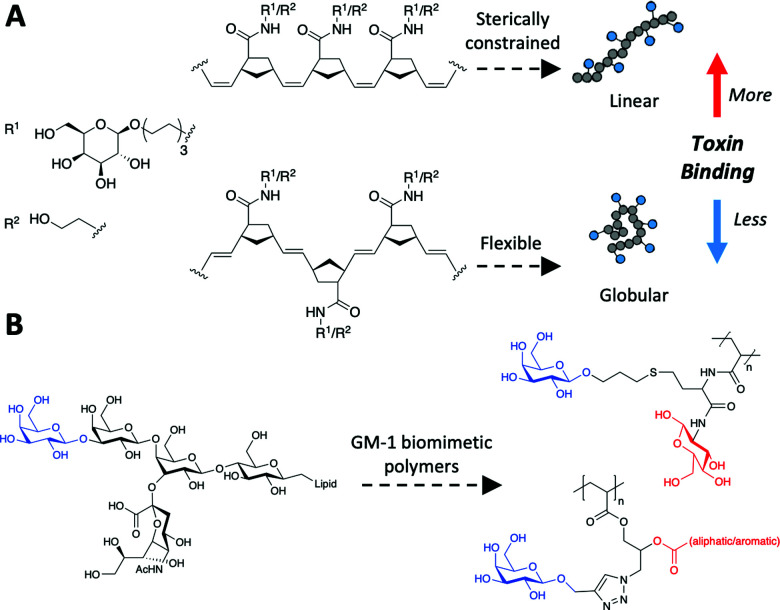
Macromolecular structure to tune cholera toxin binding. (A) control of *cis*/*trans* backbone;^177^ (B) GM-1 mimetic polymer through polymer/glycan linker modifications.^179,180^

Glycopolymers have been reported (non-exhaustively) to bind with enhanced affinity to lectins and as inhibitors of influenza,^173,174^ to sequester (and inhibit quorum sensing of) bacteria,^175^ and to prevent bacterial adhesion.^176^ It is emerging how the polymer backbone, which presents the glycans, can itself play a key role in fine-tuning affinity and selectivity. For example, Kiessling showed that *cis*-backbones (from ROMP) in mucin mimetics were more potent inhibitors than *trans* equivalents (Fig. 8A).^177^ While amide-linked mannose side-chains were found to bind ConA less than ester-linked side-chains, this difference was attributed to subtle mobility differences.^178^

Macromolecular engineering approaches have also been deployed to engineer specificity. For example, side-chain length (*i.e.* linker length between backbone and glycan) was varied to enable control over the access of galactose into the relatively deep GM1 binding pocket of the cholera toxin.^181,182^ While adding branching units on the glycan–polymer linker, the allosteric sialic acid binding site could be targeted to further increase selectivity, Fig. 8B.^179,180,183^ This approach also utilised multiple different glycans within a single polymer chain and was shown to increase affinity through a range of mechanisms^184^ beyond just targeting a second binding site – potentially steric shielding too.^185^

**Fig. 9:**
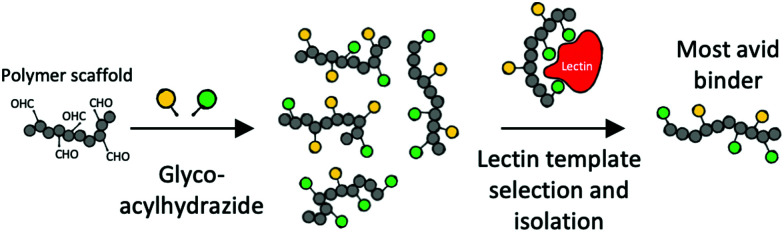
Dynamic combinatorial selection of avid lectin binders using poly-aldehydes and acylhydrazide glycans.^186^

An alternative approach to increase affinity with heterogeneous glycopolymers was introduced by Mahon *et al.* Polymer–Scaffolded Dynamic Combinatorial Libraries were used to dynamically select for high-affinity pendant groups (Fig. 9).^186^

Glycopolymers have been deployed, or show potential for, therapeutic roles and biomedical applications, including enhanced immune responses in vaccine candidates^187^ MRI contrast agents,^188,189^ drug delivery,^190,191^ anti-cancer agents^192^ and to re-program the glycocalyx.^193^ Chikae *et al.*^194^ demonstrated that glycopolymer-coated nanoparticles immobilised on carbon electrodes, can detect amyloid-β, a peptide produced in Alzheimer's disease. Others have shown the potential of glycopolymer-coated AuNPs as anti-cancer^195^ and transfection agents.^196^

Each of the above examples shows how the underpinning high-affinity binding from glycopolymers to targets has the potential to be translated into innovative biosensors and diagnostics. The structural versatility of multivalent sugar presenting systems can be used to display a glycan and precisely adjust the nature of the presentation in 3D space^197^ (as seen for native glycans) providing opportunities to tune the interaction.

## 7. Glycosylated gold nanoparticles

As discussed in the previous sections, antibody-functionalised gold nanoparticles are the most common core of lateral flow devices. There is limited literature on glyco-gold nanoparticles for LFD (discussed in the section below), but there is a broad literature on the synthesis and use of glycosylated gold nanoparticles, particularly for lectin binding, which is summarised here. Notably, gold nanoparticles are not the only nanoparticles systems used to detect lectins; examples include quantum dots and magnetic nanoparticles.^198^

Whilst many glycosylated nanoparticle syntheses report direct immobilisation, or short linkers to attach the glycan, this can lead to colloidal stability challenges and irreversible aggregation when in biologically relevant media, such as saline buffers or blood plasma. Hence, the use of polymeric tethers that provide steric stabilisation and act as non-fouling^199^ interfaces has been explored.

**Fig. 10:**
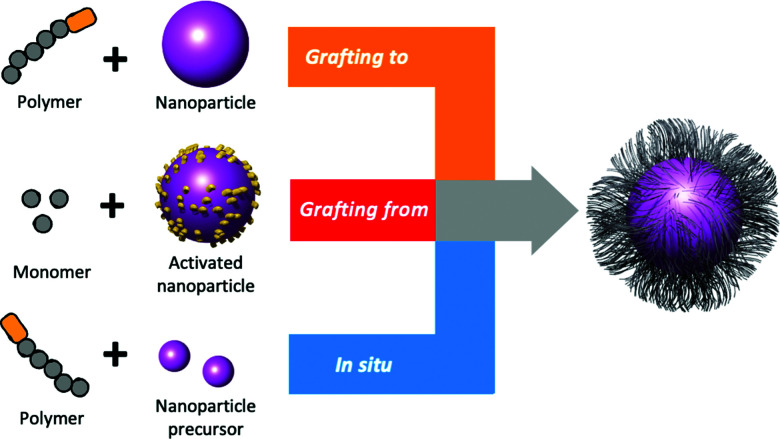
Representation of the three conceptual methods of polymer grafting to a surface.

Polymer chains can be added to the surface of AuNPs by three conceptual methods; “*grafting to*” (a polymer is added to the surface of pre-prepared AuNPs), *in situ* (a polymer is added to the surface of growing AuNPs) and “*grafting from”* (polymerisation occurs on CTAs or initiators bound to the pre-prepared AuNP surface), Fig. 10.^200^ The advantage of “*grafting to*” is that the precursor polymer can be synthesised and characterised, and installation of the glycan (or other functionality) can be confirmed, which is challenging when added directly to a nanoparticle surface. It is, however, important to note that the diffusion of a macromolecule onto the surface and steric constraints limit the grafting density. This is overcome with “*grafting from*”, which gives high densities, but it must be isolated from the surface to characterise the polymer. Finally *in situ* methods, to produce particles in the presence of a polymeric capping agent, can be used which is a balance between the two other approaches.

Poly(ethylene glycol) (PEG) is widely used as a nanoparticle coating due to its low-biofouling, low cytotoxicity and commercial availability. Russel and co-workers used a PEG linker to immobilise a tri-functional sialoside to gold nanoparticles, enabling tuning of avian *versus* human influenza binding (dictated by the 2,3 *versus* 2,6 linkages).^201^ Penadés and co-workers have used PEGylated gold nanoparticles^202^ to investigate glycosphingolipid mediated carbohydrate–carbohydrate interactions when coupled with an SPR sensor.^203^

**Fig. 11:**
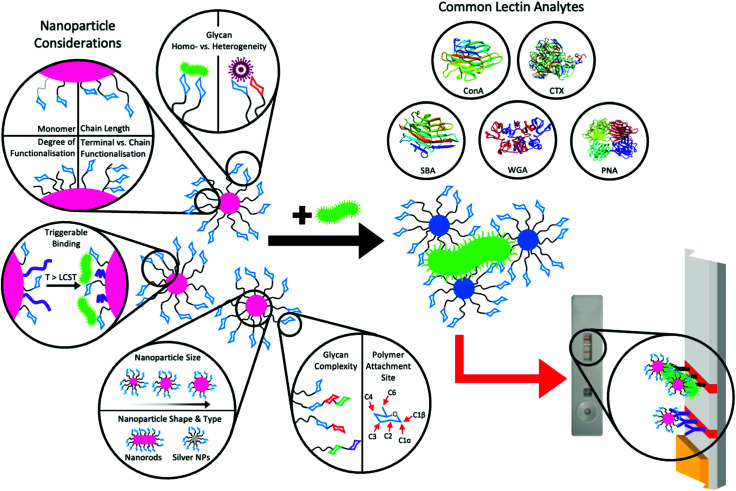
Major nanoparticle design considerations and common lectin analytes for aggregation assays and potential for use in lateral flow assays. Lectin structures were taken from the Protein Data Bank^212^ as follows; soybean agglutinin (SBA) – 1SBF,^213^ concanavalin A (ConA) – 3CNA,^214^ cholera toxin (CTX) – 1XTC,^215^ wheat germ agglutinin (WGA) – 2X52^216^ and peanut agglutinin (PNA) – 2DVD.^217^

Whilst PEG is widely used, there are thousands of other potential monomeric building blocks for the polymer tether. RAFT polymerisation has attracted interest in this respect, as it installs an α-terminal (latent) thiol on every chain, which is suitable for immobilisation onto gold surfaces.^204,205^ Cameron and co-workers used poly(galactosides) derived by RAFT for the *in situ* formation of small (<20 nm) glycosylated AuNPs, capable of lectin recognition and to generate glyco-conjugate cancer vaccines.^206^ RAFT polymerisation has also been used to make pH responsive^207^ and thermoresponsive^208^ glycopolymers on AuNPs. Other (non-RAFT) approaches using disulphide, double-headed ATRP initiators^209^ or “*grafting from*” methods have also been used.^210,211^ The versatility of these approaches enables a mix and match approach whereby polymer ligands can be applied, and the coated gold nanoparticles are easily isolated by centrifugation/washing cycles (Fig. 11).

## 8. Glycosylated AuNPs in sensors and diagnostics – from aggregation to lateral flow

Considering the large body of research on the synthesis of glycosylated materials, and integration into (gold) nanomaterials there has been obvious interest in their deployment for diagnostics and biosensing applications, such as colorimetric bioassays.^218^ When nanoparticles become localised, the surface plasmon absorption energy maximum of a plasmonic nanoparticle is altered by collective conduction-band electron oscillation *i.e.* when nanoparticles aggregate there is a strong colour change.^51,56^ Mirkin *et al.*, demonstrated that oligonucleotide-functionalised AuNPs aggregated in the presence of complementary DNA sticky ends showing how specific DNA sequences can be identified by a simple colour change in solution.^219^ Due to the fact that lectins often have multiple binding sites (*i.e.* Concanavalin A (ConA) has 4,^220^ Soybean agglutinin (SBA) has 4),^221^ multivalent glycosylated gold nanoparticles have been explored as colourimetric sensors for lectins based on red-blue colour shifts.

Many studies have focused on model (plant) lectins, such as G-rutin functionalised AuNPs (*via* a catechol) or mannosylated polymers to bind ConA,^222,223^ galactose-functionalised glycopolymers to bind *Ricinus communis* agglutinin I (RCAI or RCA_120_),^223^ galactose-functionalised glycopolymers made by copper-free click immobilised on core–shell AuNPs to bind peanut agglutinin (PNA)^224,225^ and lactose-functionalised glycopolymers to bind cholera toxin.^226^ By using polymeric linkers, nanoparticle (steric) stability in complex media is introduced, avoiding false positives and unwanted aggregation which can occur with direct-surface conjugation.^227^ The use of polymer tethers also enables additional functionality and properties, such as thermal control of glycan presentation.^228^

Toyoshima *et al.*^229^ synthesised variable density mannose- and *N*-acetylglucosamine-functionalised glycopolymers immobilised on 15, 40 and 100 nm AuNPs. Aggregation experiments were carried out with target and off-target lectins. The mannose functionalised AuNPs were assayed against ORN178 (an *E. coli* species specific for α-mannose) and aggregated.^229^ While Richards *et al.* showed how fimbrae-differing phenotypes (FimH^+^ and FimH^−^) of *E.coli* can be discriminated by glycosylated AuNPs.^230^

Aggregation is a crucial consideration of lateral flow (below) but the use of gold nanoparticles for SERS (Surface enhanced Raman scattering) for other device formats is also possible but is beyond the scope of this review. SERS has been demonstrated,^231^ for example, for both ConA^232^ (to pM levels) and human galectins.^233^

### Lateral flow

Despite the above, showing the significant body of literature on how multivalent presentation of glycans leads to large binding responses with glycosylated nanoparticles, the incorporation of these into lateral flow glyco-assays (LFGAs) has not been widely explored yet. As glycans can be chemically synthesised, there is no need to raise antibodies against emergent pathogens and the sheer range of tools to alter their presentation make them appealing targets. However, thus far there have been few examples of lateral flow glyco-assays, at the time of writing and to the best of our knowledge, this review covers all published examples (November 2021).

**Fig. 12:**
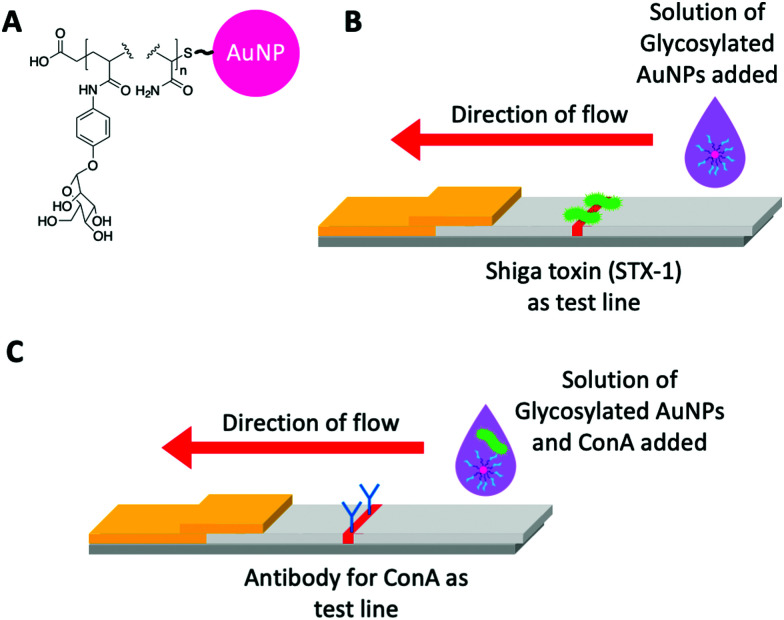
(A) Representative structure of glycosylated acrylamide coated AuNPs used by Toyoshima *et al.*^234^ and Ishii *et al.*^235^ (B) Schematic of the flow-through dipstick used by Toyoshima *et al.*^234^ and (C) Schematic of the lateral flow dipstick used by Ishii *et al.*^235^

Toyoshima *et al.*^234^ utilised α-galactose- and α-mannose- *p*-acrylamidophenyl pyranosides against lectins in aggregation assays and surface plasmon resonance (SPR) Fig. 12(A). In this work, however, they also showed a flow-through assay, where Shiga toxin-1 was deposited as the test line, facilitating some binding against the galacto–AuNPs Fig. 12(B). This was taken further by Mirua and co-workers using manno-AuNPs to detect ConA. In this case, the glyconanoparticles were used in the mobile phase and a rabbit anti-ConA antibody as the stationary phase test line Fig. 12(C). The dipsticks gave a visible ‘red line’ response to the naked eye. Whilst not a full glyco lateral flow, this study clearly demonstrated that glycoLFDs are possible.^235^ Further studies varied the mannose density providing optimisation of the output signal.^236^ A key learning point from this work, is the challenge of maintaining a good signal-to-noise ratio in the final device, with smearing of the nanoparticles as they run up the nitrocellulose due to non-specific interactions. This shows how the tuning of the polymer coating is essential alongside the use of conventional blocking agents such as BSA, to reduce non-specific interactions. The importance of the polymer coating has previously been observed in aggregation assays where a compromise must be reached between stability in aqueous media and rapid optical readout, usually tuned by adjusting polymer length.^227,230^ Similarly, alterations to the polymer itself *i.e.*, to a more sterically bulky polymer can be used to stabilise AuNPs^237^ and favour binding but avoid aggregation.^238^

**Fig. 13:**
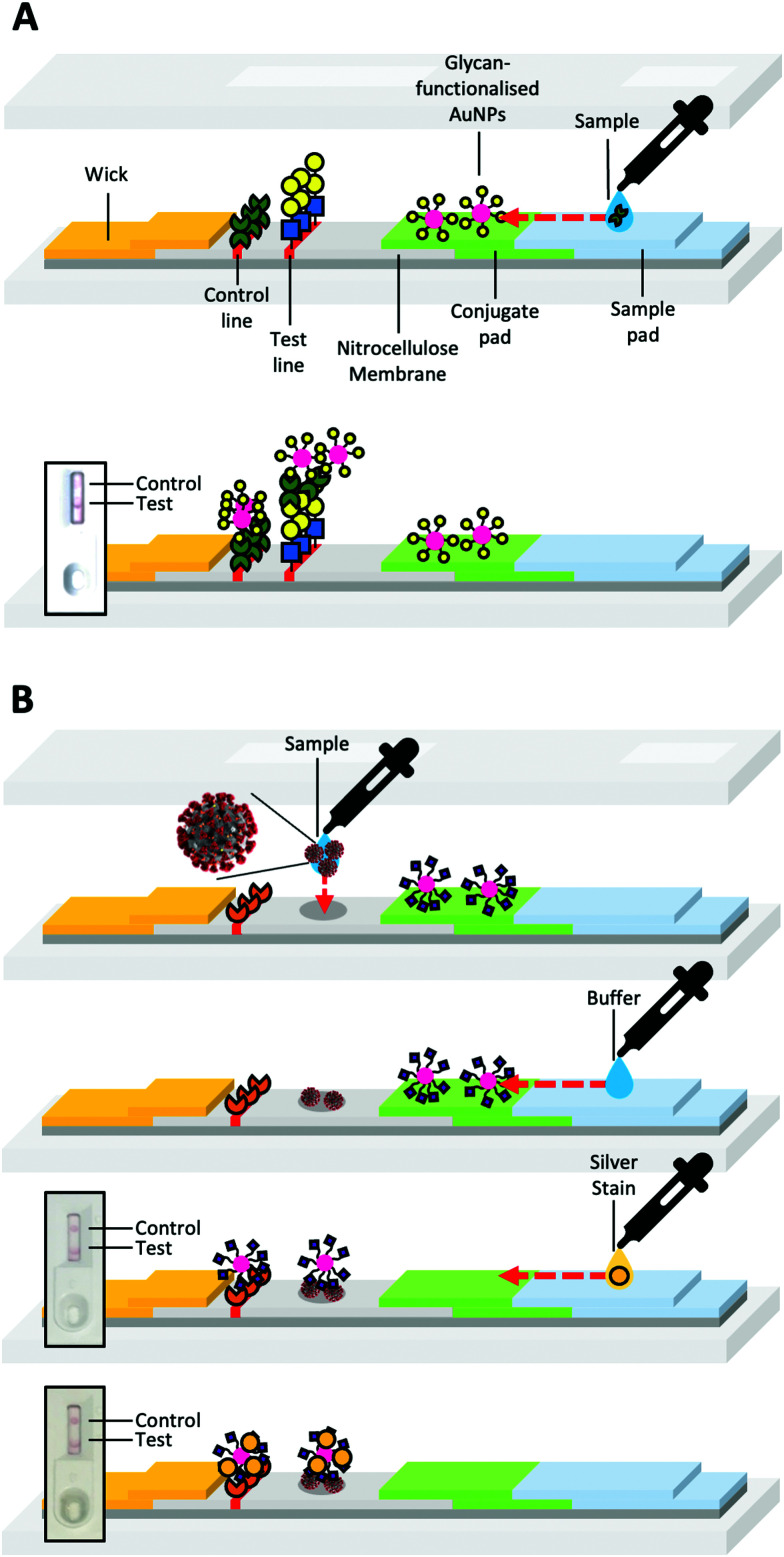
Representative lateral flow glyco-assay and flow through glyco-assay for sensing for SBA^243^ (A) and SARS-COV-2^242^ (B) respectively.

Baker *et al.*, reported that SARS-COV-2 (the causative agent of COVID-19) spike protein has affinity towards certain sialic acids,^239^ (confirmed with microarrays^240^ and STD NMR^241^) discovered using glycosylated nanoparticles against spike protein immobilised on a biolayer interferometry sensor. *N*-Acetyl neuraminic acid functionalised poly(hydroxyethyl acrylamide) were immobilised onto AuNPs and were used to detect a SARS-COV-2 spike protein bearing pseudovirus in what was the first report of a complete lateral flow glyco-assay device (using a BSA–glycoconjugate test-line).^239^ This was developed further into a flow-through device, whereby viral samples from patient swabs were dried as the test line, so no glycan test line was needed, allowing correct identification of positive/negative patients (Fig. 13A).^242^

Controlled radical polymerisation (RAFT) was employed to undertake a detailed structure-function study exploring how the polymeric glyco tether impacted the performance of a lectin-detecting lateral flow system.^243^ Using galactose as the glyco-ligand, the delicate balance between polymer chain length to give specificity, but avoid non-specific binding was revealed, demonstrated by differential binding profiles to SBA and RCA120 using the same glycan on different polymers (Fig. 13A).

Further work has also been carried out by Baker *et al.* to remove proteins from all sensing components of an LFD *i.e.*, no protein in the test line or on the particle surface. They did this by using poly(vinyl pyrrolidone) anchors decorated with glycans.^244^ This was further developed by Kim *et al.* who used glycosaminoglycans from the glycocalyx, to make a protein-free test line for SARS-COV-2.^245^ This approach of using non-protein-based polymers, both synthetic and biological, to present sugars marks a step change in LFD development and could lead to improved workflows.

## 9. Conclusions and perspectives

The purpose of this review was to introduce lateral flow diagnostic technology, including its chemical basis, and the real-world application of these devices as low-cost diagnostic tools. We then set out to show that rather than just considering these as immuno-assays, with antibodies as the detection systems, that glyco-assays are also possible by exploiting the diverse range of biochemical interactions driven by glycans. Whilst this is a very new field, with less than 10 publications, this makes it the ideal time to introduce the topic, and in particular highlight the key considerations of multivalent presentation and the importance of how glycans are tethered to the nanoparticle (*e.g.* gold) core. The potential for glycopolymer-functionalised AuNPs (or other cores) integrated into lateral flow systems, for use as POCT devices is vast, underpinned by the fast-growing field of glycomics. These low-cost, yet potentially robust glycopolymer-based lateral flow diagnostic devices could be ideal for low- and middle-income, and more economically developed countries alike.

As this is a new and exciting interdisciplinary area, spanning materials chemistry, analytical science, biochemistry, and biomedicine there exist several “challenge areas”, or areas where continued scientific exploration is needed to drive the field forward.

**Fig. 14:**
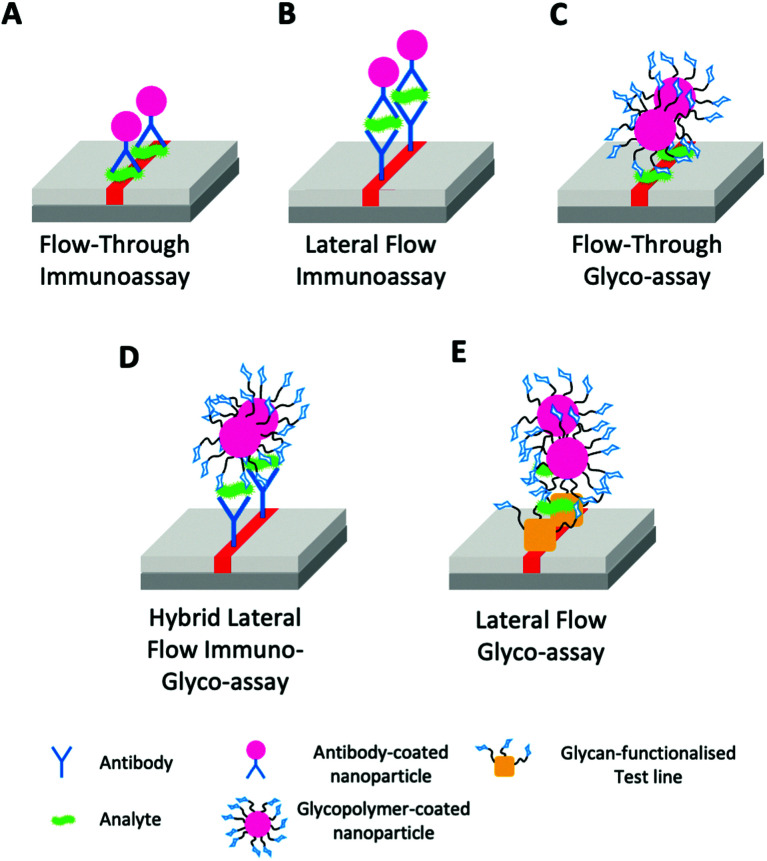
Range of lateral-flow and related assays classes highlighting the stationary phase (SP) test line and mobile phase (MP). (A) Flow-through immunoassay (SP – analyte & MP – antibody-coated AuNP); (B) Lateral flow immunoassay (SP – antibody & MP – antibody-coated AuNP and analyte); (C) Flow-through glyco-assay (SP – analyte & MP – glycan-coated AuNP); (D) Hybrid lateral flow immuno-glyco-assay (SP – antibody & MP – glycan-coated AuNP and analyte); (E) Lateral flow glyco-assay (SP – glycan-functionalised test line & MP – glycan-coated AuNP and analyte).

• The integration of new core materials, not just those based on gold or plasmonic nanoparticles.

• A greater understanding of how the linker interface impacts the performance of devices and in particular to reduce non-specific binding.

• The development of chemical tools to integrate glycans into particles more efficiently and at scale, including increasing access to rarer (often low quantity) glycans, alongside the development of the corresponding analytical tools.

• Greater integration of multiple recognition modalities and further development of multiplexed tests.

• The integration of glycan-based sensing into the various ‘flavours’ of lateral flow, or flow-through devices. (Fig. 14) both as all glyco systems, or in combination (“hybrid”) with antibodies, nanobodies, nucleic acids or other small molecule binders.

• The demonstration of function in clinically relevant situations and settings, including in complex media.

• Ensuring the devices are sufficiently sensitive and specific to be deployed for real world applications.

In summary, we have introduced the concept of lateral flow glyco-assays and their chemical basis, whilst laying out potential areas of application and the role of chemistry in developing these. It is the hope of the authors that lateral flow glyco-assays will become useful in the coming years, especially in resource-limited settings.

## Literature review methods

A review of the literature was carried out to find papers containing AuNP aggregation assays and glycopolymer use in lateral flow assays.

A review of the literature was conducted through the following databases: Web of Science (all years), Embase (1947–2021) and Scopus (all years). Search date November 2021.

The following search terms were used:

Search 1: ((gold AND nanoparticle*) OR (AuNP*)) AND glycopolymer* AND (aggregat* OR surfac*).

Search 2: (“lateral flow” OR assay*) AND glycopolymer*.

Search 3: (((gold AND nanoparticle*) OR (AuNP*)) AND glycopolymer* AND (aggregat* OR surfac*)) AND ((“lateral flow” OR assay*) AND glycopolymer*).

Search 1 returned 132 results (Embase 17, Scopus 28, Web of Science 87).

Search 2 returned 632 results (Embase 166, Scopus 221, Web of Science 245).

Search 3 returned 17 results (Embase 2, Scopus 6, Web of Science 9).

Abstracts and full-texts were screened to determine relevant reports in the literature.

## Conflicts of interest

RF is a shareholder and director of Iceni Glycoscience limited. ANB and MIG are named inventors on a patent relating to underpinning technology reported here.

## Supplementary Material
